# Advances in Nanomaterials and Composites Based on Mesoporous Materials as Antimicrobial Agents: Relevant Applications in Human Health

**DOI:** 10.3390/antibiotics13020173

**Published:** 2024-02-09

**Authors:** Germán E. Gomez, Mariana Hamer, Matías D. Regiart, Gonzalo R. Tortella, Amedea B. Seabra, Galo J. A. A. Soler Illia, Martín A. Fernández-Baldo

**Affiliations:** 1 Instituto de Investigaciones en Tecnología Química (INTEQUI), Departamento de Química, Universidad Nacional de San Luis (UNSL), CONICET, Ejército de los Andes 950, San Luis D5700BWS, Argentina; 2 Instituto de Ciencias, Universidad Nacional de General Sarmiento-CONICET, Juan María Gutiérrez 1150, Los Polvorines CP1613, Argentina; mhamer@campus.ungs.edu.ar; 3 Instituto de Química San Luis (INQUISAL), Departamento de Química, Universidad Nacional de San Luis (UNSL), CONICET, Chacabuco 917, San Luis D5700BWS, Argentina; mregiart@unsl.edu.ar; 4 Centro de Excelencia en Investigación Biotecnológica Aplicada al Medio Ambiente (CIBAMA), Facultad de Ingeniería y Ciencias, Universidad de La Frontera, Av. Francisco Salazar 01145, Temuco 4811230, Chile; 5 Departamento de Ingeniería Química, Facultad de Ingeniería y Ciencias, Universidad de La Frontera, Av. Francisco Salazar 01145, Temuco 4811230, Chile; 6 Center for Natural and Human Sciences, Federal University of ABC (UFABC), Avenida dos Estados, Saint Andrew 09210-580, Brazil; amedea.seabra@ufabc.edu.br; 7 Instituto de Nanosistemas, Escuela de Bio y Nanotecnología, Universidad Nacional de General San Martín-CONICET, Av. 25 de mayo 1169, San Martín B1650KNA, Argentina; gsoler-illia@unsam.edu.ar

**Keywords:** nanomaterials, MOFs, mesoporous materials, antimicrobial agents, biomedical applications

## Abstract

Nanotechnology has emerged as a cornerstone in contemporary research, marked by the advent of advanced technologies aimed at nanoengineering materials with diverse applications, particularly to address challenges in human health. Among these challenges, antimicrobial resistance (AMR) has risen as a significant and pressing threat to public health, creating obstacles in preventing and treating persistent diseases. Despite efforts in recent decades to combat AMR, global trends indicate an ongoing and concerning increase in AMR. The primary contributors to the escalation of AMR are the misuse and overuse of various antimicrobial agents in healthcare settings. This has led to severe consequences not only in terms of compromised treatment outcomes but also in terms of substantial financial burdens. The economic impact of AMR is reflected in skyrocketing healthcare costs attributed to heightened hospital admissions and increased drug usage. To address this critical issue, it is imperative to implement effective strategies for antimicrobial therapies. This comprehensive review will explore the latest scientific breakthroughs within the metal–organic frameworks and the use of mesoporous metallic oxide derivates as antimicrobial agents. We will explore their biomedical applications in human health, shedding light on promising avenues for combating AMR. Finally, we will conclude the current state of research and offer perspectives on the future development of these nanomaterials in the ongoing battle against AMR.

## 1. Introduction

In the last decade, there has been a notable increase in the consequences of antimicrobial resistance (AMR). This often implies the need for higher doses of drugs and multiple treatments over a prolonged period to treat various microbial infections [1]. Consequently, it conduces to high toxicity, secondary effects, and an increment in the therapy cost. Currently, AMR is one of the main concerns worldwide [2]. The preoccupation with new resistance mechanisms of pathogen microorganisms continues to threaten our ability to treat common infections [2]. A frequent problem is the rapid propagation of multi- and panresistant bacteria, which cannot be treated using common antibiotics [3]. In addition, greater inversion is required to research and develop new antimicrobial agents, especially those targeting critical Gram-negative bacteria and other pathogenic microorganisms such as resistant ones. To address these problems, new technologies such as nanotechnology and nano-enabled therapies are urgently needed to combat pathogenic microorganisms that cause human infections and overcome multidrug resistance [3].

Recently, the use of nanomaterials in antibiotic formulations has been promising. Diverse mesoporous materials (MMs), such as metal–organic frameworks (MOFs) [4], mesoporous silica nanoparticles (MSNs) [5], and titania thin films (MTNs) [6,7], among others, have demonstrated interesting antimicrobial properties. Furthermore, the mesoporous characteristics, including large surface area, confinement effect, and chemical and crystalline structure stabilities exhibited by the mentioned systems, make it plausible to design synthesis strategies, functionalization, miniaturization, and deposition, and devise implementations for biocide applications.

MOF materials are emerging porous materials that have demonstrated a high potential for antimicrobial therapy [8,9,10,11,12,13]. These materials are constructed by forming coordination bonds between metal nodes and organic linkers [10,11,12,13]. Generally, MOFs are compounds with strong bonds between metal inorganic subunits and organic ligands. Many combinations of metal and organic linkers exist to form various MOFs with interesting medical applications. Some advantages of MOFs over other nanomaterials are their high surface area, tunable and uniform porous structure, flexible, functional metal sites and organic groups across the entire network, and high designability [14].

Furthermore, properties such as the geometry, structure, composition, pore size, surface area, and surface functionality of MOFs can be flexibly and rationally tuned by changing the combination of organic and inorganic components. Also, there are three principal antimicrobial mechanisms of MOFs [15]. First, metal ions with antimicrobial power, such as silver, zinc, and copper, can be released from the MOF structure and inhibit the growth of microorganisms. Furthermore, an interesting aspect is that the organic ligands of the structure could behave as antibiotics or photosensitizers, which can generate ROS to kill bacteria through light irradiation. Finally, these materials could act as carriers to load antibacterial agents such as antibiotics, photosensitizers, or antimicrobial peptides, among others, to encourage antibacterial efficacy [14,15].

On the other hand, MMs are compounds that present a wide range of medical applications. MM materials are synthesized using, for example, organic surfactants, and they have well-ordered pores, but the pore walls are frequently amorphous [16,17]. These materials have a pore size from 2 to 50 nm (IUPAC). Mesoporous metallic oxide derivates present a plethora of potential applications in bio-applications, including bone tissue engineering and preparation, multimodal imaging (i.e., luminescence, contrast agents), theranostics, or delivery of molecules of pharmacological relevance [18,19]. Exploiting the structural and textural properties of MMs allows hosting and potentially delivering of a wide range of therapeutic applications such as in health human treatment (cancer, osteoporosis, and infection) [20,21]. In addition, the texture of mesoporous thin films with well-defined pore sizes between 4 and 7 nm was shown to affect cell growth and proliferation; mesoporous silica films have the desired properties in reducing biofilm formation and bacterial attachment for relevant biomedical applications [22,23]. These unique features make mesoporous nanomaterials an ideal nanoplatform for bacterial infection treatment [24,25]. 

Furthermore, in 2021, Gomez and Soler Illia [26] published a virtual issue focusing on recent developments in multifunctional nanoporous materials, encompassing MOFs and metallic oxide derivates. These materials possess highly tunable porosity, functionalization adaptability, particle size reduction feasibility, and ease of processing and manipulation for device construction. These qualities render both types of materials well-suited for use as antimicrobial agents.

This review will concentrate on the latest scientific progress in utilizing MOFs and their derivatives, along with MMs, as antimicrobial agents and for biomedical applications. A subsequent discussion will explore their advantages in promoting human health. In conclusion, we will summarize trends and offer insights into the prospects for developing these nanomaterials.

## 2. MOF Materials as Antimicrobial Agents: Applications in Human Health

**Figure 1:**
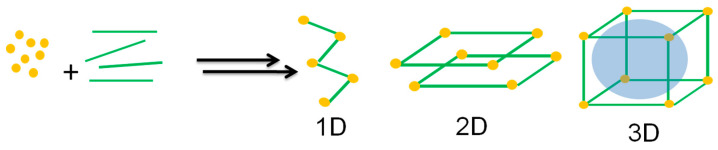
Self-assembly process towards MOF synthesis. Figure made by the authors.

According to the International Union of Pure and Applied Chemistry (IUPAC) [27], a coordination polymer is a compound with repeating inorganic moieties extending in one, two, or three dimensions. In particular, the polymer can extend in two or three dimensions in a coordination network. In addition, in an MOF, organic ligands coordinate the inorganic ions or clusters that form supramolecular structures containing potential cavities [27,28,29,30]. Moreover, it is possible to combine organic ligands and various metal ions to modify their surface area, structures, pore size, and functionalities for promising applications such as selective separation and gas storage, drug delivery, catalysis, sensing, and optoelectronic device implementation [31]. Figure 1 depicts the self-assembly process for obtaining MOF materials. 

**Figure 2:**
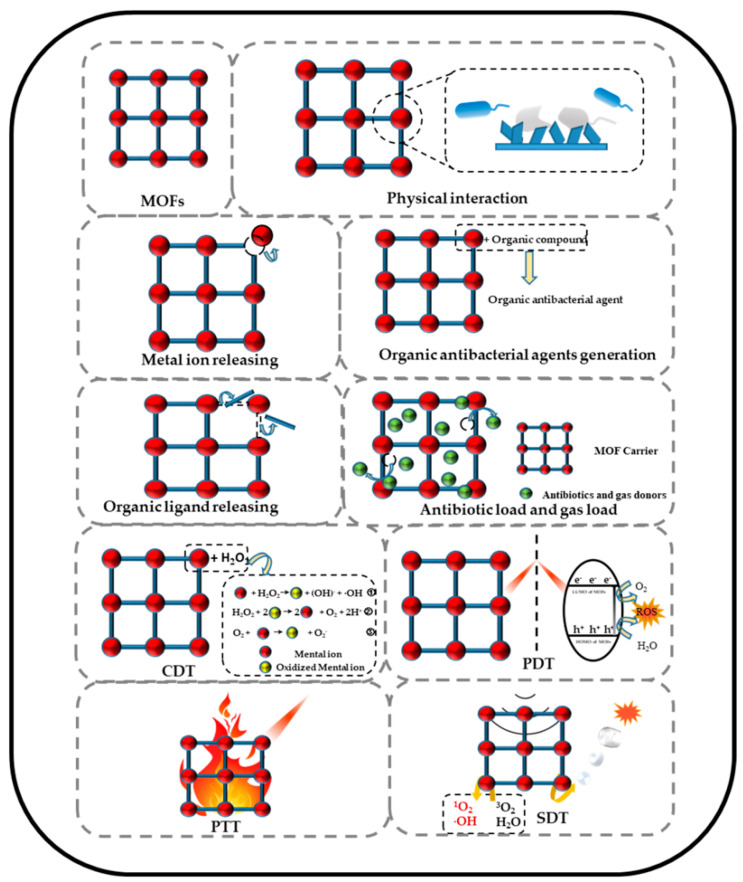
Schematic illustration of MOFs’ antibacterial mechanisms. Reproduced from reference Zang et al. [15], 2022, under the terms and conditions of the Creative Commons Attribution (CC BY) license. https://doi.org/10.3390/jfb13040215.

Moreover, in recent years, various mechanisms have undergone thorough examination, with a noteworthy increase in articles addressing the eradication of bacterial species, allowing the publication of recent reviews. Zang et al. [15] documented diverse MOF materials exhibiting antibacterial activity. These materials operate through action mechanisms that encompass physical interaction (PI), organic ligand releasing (OLR), metal ion releasing (MIR), gas and antibiotic releasing, chemical dynamic (CDT), photodynamic (PDT), photothermal (PTT), synergistic, and sonodynamic therapy (SDT) (see Figure 2). Consequently, this review aims to spotlight recent advancements in this field. Various MOFs have been thoroughly researched due to their favorable antibacterial effects, which stem from distinct chemical and physical attributes, such as the gradual release of organic compounds or metal ions and their photocatalytic, enzyme-like, ultrasonic, and photothermal activities. An excellent review has treated the action mechanisms [15], which is not the purpose of the present revision.

Significant revisions in the MOFs’ antimicrobial activities have emerged in the last five years. In 2022, Dai and colleagues [32] provided a comprehensive and well-described summary of significant advances in the silver–MOF and silver–MOF composites’ performance for bacterial eradication. Marchetti et al. [8] also presented an excellent compilation highlighting the most relevant cases of transition-metal-based MOF composites utilized as antibacterial, antiviral, and antifungal agents against a diverse range of microbes. These contributions assist our understanding of the potential applications of MOFs in combating microbial threats.

**Figure 3:**
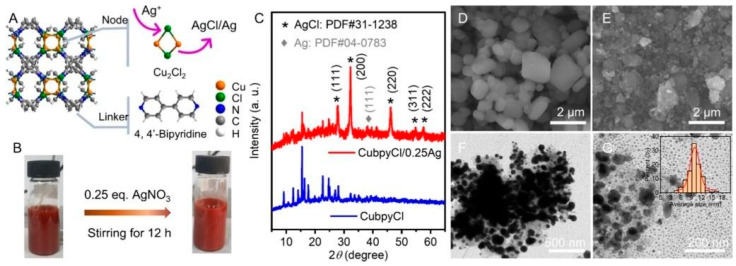
Diagram of CuBpyCl/Ag compound (**A**), preparation of the CuBpyCl/Ag material (**B**), XRD characterizations (**C**), and (**D**,**E**,**F** and **G**) SEM/TEM analysis of CuBPyCl/Ag NPs. Reprinted with permission from Mao et al. [33]. Copyright 2023 American Chemical Society.

**Figure 4:**
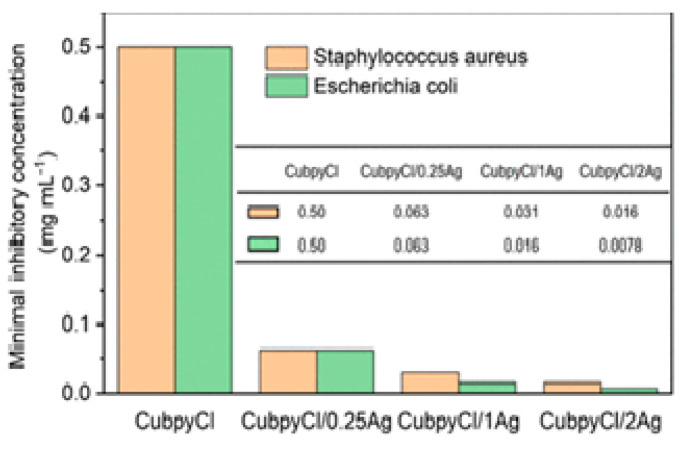
CuBPyCl/Agb minimum inhibitory concentration and sensitivities values against *S. Aureus* and *E. Coli*. Reprinted with permission from Mao et al. [33]. Copyright 2023 American Chemical Society.

Silver NPs have proven to be a successful method for eradicating bacterial populations [32]. In the field of MOFs, incorporating metallic NPs into porous materials through post-synthetic procedures is relatively straightforward. A study by Mao et al. [33] showed the feasibility of introducing AgCl/Ag NPs into a 3D porous Cu-MOF, such as Cu(I)bpyCl (bpy = 4,4′-bipyridine), targeting *S. Aureus* and *E. coli*. The NPs were synthesized using a simple “one-pot” methodology, resulting in a crystalline size of approximately 10 nm, and their structure was characterized using X-ray diffraction (see Figure 3C). The silver-functionalized samples exhibited lower minimum inhibitory concentration (MIC) values (16 µg L^−1^ against *S. Aureus* and 7.8 µg L^−1^ against *E. coli*) compared to Cu-MOF samples without NPs (see Figure 4).

In addition, the interest in developing new antimicrobials led to new alternative treatments for infections caused by pathogenic microbes. Photodynamic inactivation seems to be a suitable technique. To accomplish this, sensitizing photoactive substances are irradiated at specific wavelengths in the presence of oxygen to produce ROS through one of two photochemical reactions, electron transfer (type I) or energy transfer (type II), causing cell damage and death of pathogenic microorganisms. Godoy et al. [34] obtained nanosized particles of the Tb-doped Ysucc-sal compound by employing a cationic surfactant as a bottom-up approach to modulate the two 2D coordination networks’ particle sizes. In addition, combined with the top-down technique, the delamination of crystals was implemented to produce nanosized systems. One reported sample showed photoinactivation of *C. albicans* at different proportions, reaching almost 100% microbe inhibition. Also, singlet oxygen was confirmed as the principal species for photoinactivation of *C. albicans* with these systems. This work was valuable and needed to develop new photoactive compounds for topical formulations. 

**Figure 5:**
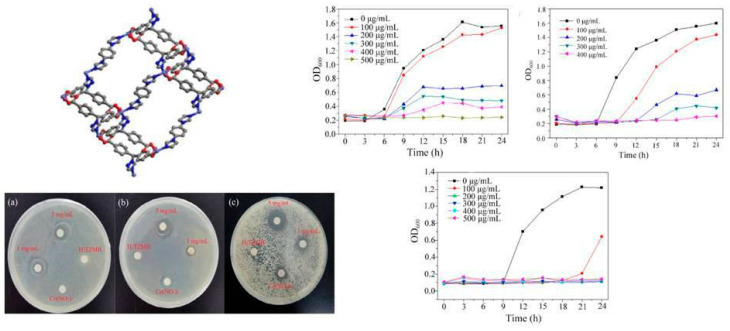
[Co_2_(TZMB)2(1,4-bib)_0.5_(H_2_O)_2_]·2H_2_O crystalline structure. MOF inhibition zone against (**a**) *S. aureus*, (**b**) *E. coli*, and (**c**) *C. albicans*. Right: MOF growth curve against (**a**) *S. aureus*, (**b**) *E. coli*, and (**c**) *C. albicans.* Reproduced with modification from reference Jing et al. [35], 2023, under the terms and conditions of the Creative Commons Attribution (CC BY) license. https://doi.org/10.3390/molecules28135204.

A recent multifunctional 3D-MOF example, [Co_2_(TZMB)_2_(1,4-bib)_0.5_(H_2_O)_2_]·2H_2_O (Figure 5), was prepared. Also, properties like dye adsorption and antibacterial and photocatalytic activity were explored [35]. Control experiments revealed that metal salts and mixed ligands had no antibacterial effect on the bacterial solution. At a 5 mg mL^−1^ concentration, the MOF exhibited a bacteriostatic circle diameter of 14 mm, with a 500 μg mL^−1^ MIC for *S. aureus* (Figure 5), 11 mm and 400 μg mL^−1^ MIC for *E. coli*, and for *C. albicans*, they were 11 mm and 200 μg mL^−1^, respectively. Notably, the compound demonstrated complete growth inhibition for all microorganisms within 24 h at 400–500 μg mL^−1^ suspension concentrations (Figure 5).

**Figure 6:**
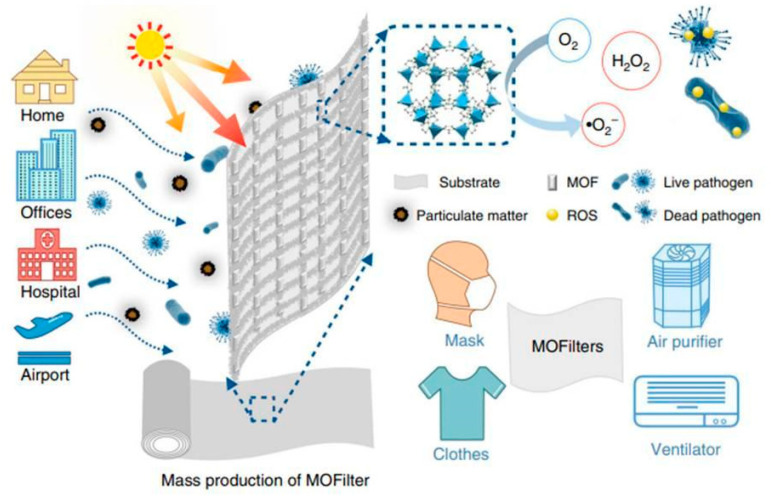
Scheme of MOF-based filters for integrated air cleaning. Reproduced from reference Li et al. [36], 2019, under the terms and conditions of the Creative Commons Attribution (CC BY) license. https://doi.org/10.1038/s41467-019-10218-9.

**Figure 7:**
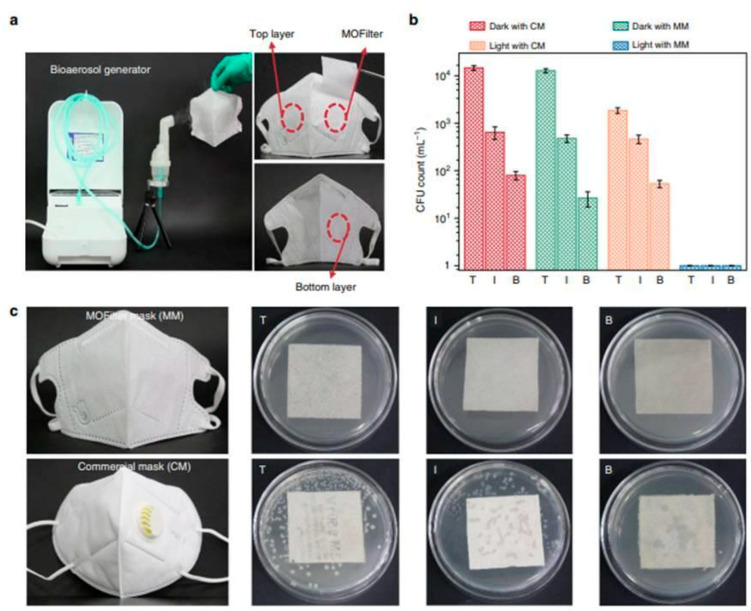
Comparison of the antibacterial performance between MOF filter mask (MM) and commercial mask (CM). (**a**) Bioaerosol generation apparatus and optical images of trilaminar MOF filter masks. (**b**) *E.coli* levels residual on top (T), inner (I), and bottom (B) layers of MM and CM after 30 min of light irradiation, respectively. (**c**) Bacterial colonies residual on eluent-treated masks. Reproduced from reference Li et al. [36], 2019, under the terms and conditions of the Creative Commons Attribution (CC BY) license. https://doi.org/10.1038/s41467-019-10218-9.

As mentioned earlier, MOF composites offer valuable examples of innovative technological applications, and many immobilization techniques enable the use of MOF systems as devices for various purposes. Recent examples have garnered attention in antimicrobial materials, particularly integrating MOFs into cotton fibers to develop personal protective clothing (see Figure 6). Under solar photocatalytic conditions, MOF@fiber composites were developed as active pathogen inhibitors. Li et al. [36] reported the implementation of nanosized ZIF-8 (zeolitic imidazolate frameworks) produced by the aerosol method and further impregnation into fibers (Figure 7). In addition, the Zn^2+^ charge trapping centers can be photogenerated on the MOF surface via ligand-to-metal charge transfer (LMCT) and active O_2_ to form ^•^O_2_− and related ROS like H_2_O_2_. ROS production mainly contributes to the ZIF-8 biocidal properties. So, ZIF-8 is a filter with a synergic bacteria-killing and particulate matter filtration performance. It was demonstrated that ZIF-8-based filters could protect against pathogens, aerosols, and air hazards. 

**Figure 8:**
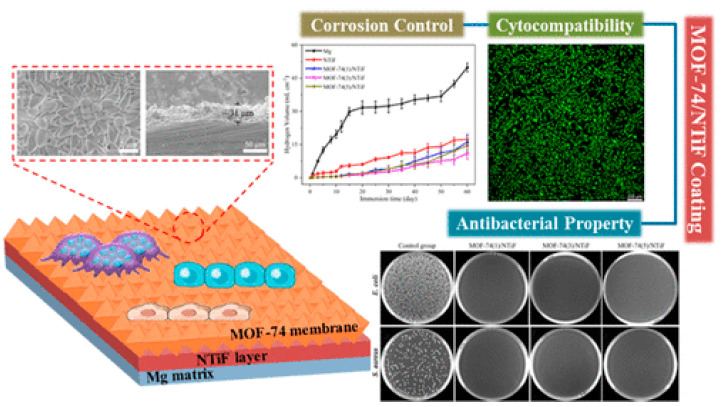
Membranes constructed by MOF-74/NTiF exhibit exceptional antibacterial, anticorrosion, and cytocompatibility properties. Reprinted with permission from Liu et al. [37]. Copyright 2023 American Chemical Society.

MOF-74 is a well-known 3D-MOF structure based on Zn^2+^ ions connected by 2,5-dihydroxyterephthalate linkers. Recently, MOF-74-based membranes on an NH_4_TiOF_3_ (NTiF) layer-modified Mg matrix were prepared for antibacterial, cytocompatibility, and corrosion control properties [37]. Corrosion protection was enhanced by outer MOF-74 membranes whose crystals and thicknesses could be adjusted for several protective effects. Also, MOF-74 membranes promoted proliferation and cell adhesion, showing outstanding cytocompatibility. Using MOF-74 decomposition to generate the Zn^2+^ and 2,5-dihydroxyterephthalate liberation could effectively inhibit *S. aureus* and *E. coli*, showing highly efficient antibacterial properties (see Figure 8).

More recently, a novel synthetic laser ablation method was used to obtain MOF-5/graphene composites as novel platforms for bacterial inhibition [38]. MOF-5, known as Zn-terephthalate, is one of the most explored materials in the MOF field. The nanosheets were employed as antibacterial agents against *S. aureus* and *E. coli*, accompanied by around 0.125 μg mL^−1^ MIC values.

Duncan et al. [39] demonstrated that CPO-27 (Ni), a 3D-MOF constructed by divalent nickel ions connected by 2,5-dihydroxyterephtalate linkers, can be synthesized into polyurethane films using solvent-casting methods. Also, the films can be activated and loaded with NO gas. Antibacterial NO concentrations were released when in contact with a moist environment, and antibacterial performance against *S. aureus* and *E. coli* can be achieved within 1 contact hour employing 5 wt% MOF films. These studies advance the understanding of NO-releasing MOFs for medical applications.

Bismuth has an extensive history of medicinal use and applications in medicine and healthcare, including treating gastrointestinal disorders, antitumor activities, and antimicrobial and antibacterial properties [40]. In this context, Bi-MOFs have been obtained in the last decade with potential applications in bacterial eradication. Remarkably, our group reported the strong antibacterial effect of the two Bi-MOFs on *E. coli*, *Typhimurium*, *S. Typhimurium*, and *P. aeruginosa* compared to the bismuth salt, Bi-citrate [41]. Moreover, Huang and colleagues [42] recently reported two rapid synthesis methods, ultrasound-assisted and agitation-free, to produce bismuth-based materials with ellipsoid and rod-like nanomorphologies, respectively. The porous structure of rods was identified as Bi-MOFs, aligning with the crystalline structure of CAU-17. CAU-17 is a bismuth-based MOF, [Bi(BTC)(H_2_O)]·2H_2_O·MeOH, with 1,3,5-benzenetricarbolylate as linkers. In addition, ellipsoids and rods demonstrated excellent biocompatibility with human gingival fibroblasts and exhibited high antimicrobial effects against Gram-negative oral pathogens, including *Aggregatibacter actinomycetemcomitans*, *Porphyromonas gingivalis*, and *Fusobacterium nucleatum*. At a concentration of 50 μg/mL, both ellipsoids and rods were able to disrupt bacterial membranes, with particular efficacy in eliminating *P. gingivalis* biofilms. This suggests promising applications for these bismuth-based materials in combating oral pathogens while remaining biocompatible with human cells.

In addition, immobilizing MOF nanoparticles and other additives into membranes is an excellent option for constructing technological devices for antibacterial applications. Nevertheless, there is a scarce number of reports of antiviral applications of MOFs, mostly in polymeric matrices. Gupta et al. [43] have recently reported immobilizing HKUST nanoparticles and carbon nanotubes (CNTs) into PVDF filtration membranes, demonstrating a decrease in the virus population by 80%. Another recent example of immobilized MOF into polymeric membranes is the reported case by Lee et al. [44], where a PDMS@Cu-MOF composite was obtained, exhibiting antibacterial activity against *E. coli*, *S. aureus*, *P. aeruginosa*, *K. pneumoniae*, and methicillin-resistant *S. aureus (MRSA)*. 

As exemplified, composite construction seems to be an excellent way to increment the antimicrobial activity of MOF materials. However, the formulation of drug@MOF systems could be a novel scenario for the capture and further controlled liberation into liquid and solid mediums for microbial eradication. In this context, Shakir and colleagues [45] reported a levoflaxin@Zn-MOF against Gram-positive and Gram-negative bacteria, specifically *S. aureus* (MIC-64.4 μg mL^−1^), *B. subtilis* (MIC-94.97 μg mL^−1^), *E. coli* (MIC-26.0 μg mL^−1^), and *P. aeruginosa* (MIC-67.48 μg mL^−1^).

On the other hand, over the past few years, there has been a growing interest in implementing MOFs for biomedical purposes, particularly in drug delivery. As MOF particles have been miniaturized to the nanoscale, these diminutive structures, known as nano-MOFs, have emerged as highly effective nanocarriers capable of delivering various agents for applications such as imaging, chemotherapy, photothermal therapy, and photodynamic therapy due to their adjustable aperture, large surface area, large pore capacity, controllable drug release, and easy modification [46]. However, to fulfill pharmacological and biological prerequisites, MOFs utilized as nanocarriers for antimicrobial purposes must exhibit the following critical attributes: (i) precise control of drug release, avoiding an initial burst; (ii) exceptional drug-loading capacity; (iii) customizable surface for targeted therapy; and (vi) absence of cytotoxicity. Consequently, choosing suitable materials for encapsulating various antimicrobial active substances should align with these specified properties [47,48,49].

Also, Desai et al. [50] successfully introduced highly porous MOFs as promising materials to combat the transmission of airborne viruses, including coronaviruses. The post-synthetic modified Zr(IV)-based UiO-66 MOFs (UiO-66, UiO-66-NH_2_, and UiO-66-NO_2_), with three antiviral agents—namely, nystatin, folic acid, and tenofovir—proved to be remarkably effective in enhancing the binding affinity toward various proteins such as glycosylated BSA, nonglycosylated Annexin-g03104, and the spike protein of SARS. Additionally, the internal pores of the MOFs remain accessible to water even after functionalization, which is expected to induce local dehydration and deactivate viruses. The UiO-66 series is a known family of 3D-MOFs constructed by zirconium oxo-clusters linked by benzedicarboxylate linkers. This “proof-of-concept” study sets the stage for further investigations involving diverse functional drug molecules and MOF structures and their combination with fabrics.

MOFs have demonstrated the capability to incorporate antibacterial compounds, including antibiotics, metals, metal oxides, natural plant products, and nitric oxide, within their frameworks through different mechanisms [49]. Antimicrobial agents can be adsorbed in the MOF surfaces, absorbed inside the pores, immobilized through covalent binding, and used as building blocks to construct the crystalline structure. Consequently, precise control over drug release can be achieved by adjusting MOFs’ porosity, biodegradability, and external stimulus conditions, such as light and pH. Furthermore, the degradation of MOFs can also result in the release of metal ions, further enhancing their antimicrobial effect through synergy [4,47,49,50].

Table 1 depicts some examples of MOF-based carriers for antimicrobial application, their building block components, the antibacterial agent, the drug release, and the pathogenic agent in which their activity was tested.

## 3. MMs Based on Silicon and Titanium Derivates as Antimicrobial Agents

**Figure 9:**
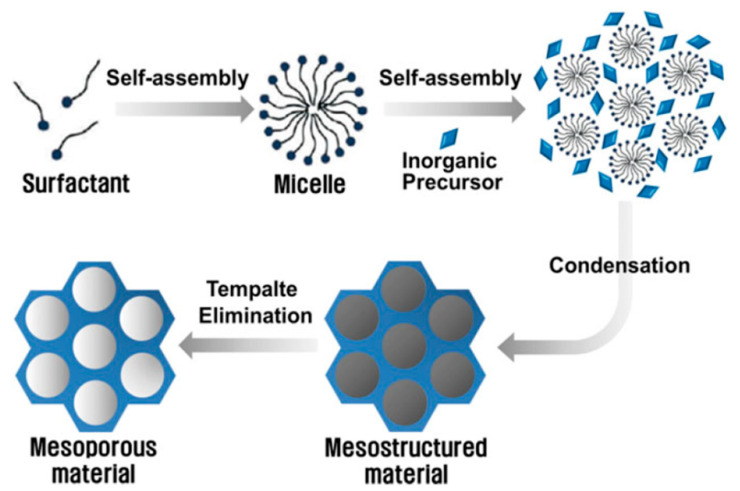
Self-assembly process of Si and Ti MMs reported by Ha and Park [63].

Over the past few decades, diverse chemical approaches have been employed to create templated MMs with organized arrays of mesopores [61,62]. Numerous articles focus on controlling the sizes and shapes of pores in MMs. Various synthetic methods, including soft templating, hard templating, nanocasting, electrochemical techniques, surface functionalization, and the entrapment of species within pores, have been utilized to regulate porosity. Figure 9 outlines the more common process to obtain MMs based on Si and Ti ions. The precursors used for synthesizing usually consist of a metal or metalloid element whose reactivity is controlled by the reaction conditions or ligands, and typically, surfactants are used as pore templates [63].

Interestingly, the mesoporous texture influences the adhesion and growth of cells and bacterial colonies. For example, osteoblasts develop differently in mesostructured TiO_2_ surfaces, concerning plain glass; this was associated with the increased formation of filopodia, resulting in higher cell adhesion, proliferation, and viability for nonmodified glass slides [64]. A detailed study on osteoblast growth on mesoporous oxide thin films demonstrated that both the inorganic matrix and the pore diameter affect cell adhesion and proliferation. While in short periods (3 to 6 h), cell–surface interactions are governed by the surface hydrophilic/hydrophobic properties, in longer terms (i.e., days), the nanotopography and surface chemistry affect osteoblast adhesion and proliferation; larger mesopores lead to slower growth and lower cell density [22]. This nanotopographical growth control concept was applied successfully to arrest biofilm formation. Indeed, templated mesoporous coatings inhibit the formation of *P. aeruginosa* biofilms in submerged or air–liquid interface conditions. Experiments performed on variable mesopore size demonstrate that mesoporous silica coatings with larger pore size significantly reduce the number of colonies and avoid the formation of a biofilm matrix, facilitating bacteria elimination and leading to transparent and robust antibiofilm coatings [23,65]. Titania mesoporous thin films combine the possibility of avoiding biofilm formation and enhancing the bactericidal UVA effect on *P. aeruginosa* by five orders of magnitude through the photocatalytic effect [66].

In the last few years, multiple nanocomposite materials based on mesoporous silicon and silica materials with antimicrobial activity have been developed for biomedical devices. Recently, Sviridov et al. [67] created mesoporous silicon nanoparticles intended for treating purulent wounds and for antibacterial cleansing of various aqueous suspensions and biological fluids. The nanoparticles were produced through electrochemical etching of crystalline silicon wafers, followed by high-energy milling in water. On the other hand, in vitro experiments with *L. casei* demonstrated a significant reduction in bacterial viability and cell damage upon exposure to ultrasonic irradiation in the presence of nanoparticles.

**Figure 10:**
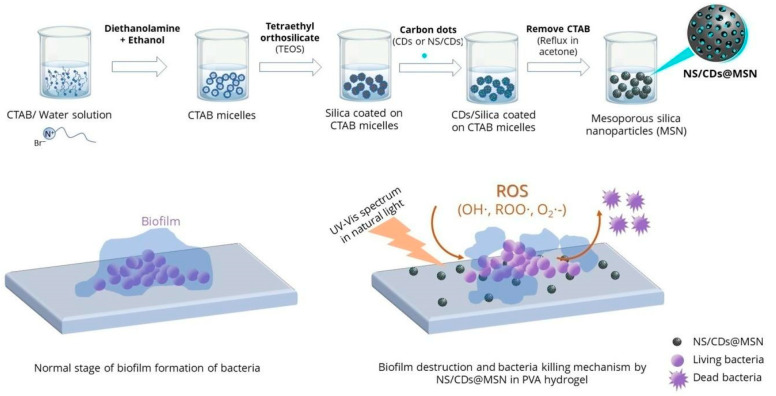
Schematic representation of the preparation of MSNs and CDs@MSN or NS/CDs@MSN nanocomposites and their antimicrobial activity. Reproduced from reference Pongchaikul et al. [69], 2023, under the terms and conditions of the Creative Commons Attribution (CC BY) license. https://doi.org/10.1016/j.ijpx.2023.100209.

Multiple methodologies are used to increase or enhance the antimicrobial effect in mesoporous silica materials. An example is incorporating bactericidal species in the surfaces or mesopores, which generates an increased synergistic effect. One of the most straightforward systems is a mesostructured F127-templated titania coating, in which the trapped template reveals itself as a highly bactericidal coating (effectivity above 99.9%) against five clinically significant Gram-negative and Gram-positive pathogens. It was proposed that a synergy between the topographical arrangement and the nanopolymer at the mesostructured Pluronic–titania interface can harm bacterial cell wall integrity [68]. In another development, a nanocomposite hydrogel that effectively controls biofilm and has antimicrobial properties for application to heal persistent or long-lasting wounds [69] was developed. Core/shell mesoporous silica nanostructures (MSNs) and carbon dots (CDs) were developed to exhibit antimicrobial activity against *S. aureus*, *P. aeruginosa*, and *E. coli*. Integrating nitrogen- and sulfur-doped CDs (NS/CDs) with MSNs demonstrated antimicrobial activity four times higher than silver nanoparticles (AgNPs). Specifically, NS/CDs@MSN nanoparticles displayed superior minimal biofilm inhibitory effects at low concentrations (<0.125 mg/mL). These nanostructures were incorporated into polyvinyl alcohol (PVA) hydrogel, and their antimicrobial efficacy, biofilm control, and cytotoxicity toward fibroblast cells were evaluated for potential biomedical applications. Figure 10 shows a schematic representation of the preparation of MSN and CDs@MSN or NS/CDs@MSN nanocomposites, along with their antimicrobial activity [69].

The most extended use of mesoporous silica for antibacterials is undoubtedly the loading with silver species, which have been widely used in a synergic way due to their known antimicrobial activity. For example, Catalano et al. [70] developed continuous silica and titania mesoporous thin films deposited on glass containing AgNPs or adsorbed Ag^+^ ions in the mesopores. These coatings present extreme bactericidal efficiency against *P. aeruginosa* and *S. aureus*. In addition, films are robust and can withstand at least ten cycles of use, demonstrating residual action. In addition, films loaded with silver cations are optically transparent while keeping long-lasting antibacterial activity, which makes them attractive for architectonics or sanitary applications.

Further, a combination of Ag^+^ ions and other antibiotics was loaded into functionalized SBA-15 or mesoporous silica nanoparticles, which were then well dispersed in a multiphase system. The resultant emulsion can be readily administered via spray coating on any surface, ensuring swift surface disinfection and providing lasting protection against microbial growth for 24 to 72 h. The coating was effective towards various microorganisms (*E. coli*, *P. aeruginosa*, *S. choleraesuis*, *B. cereus*, and *S. aureus*), and Coronaviridae. These performances and figures of merit comply with the disinfection standards of health institution protocols. The technology developed by Hybridon-ADOX has been approved by health authorities in Argentina and has been available for commercialization since 2023 [71].

Montalvo-Quirós et al. [68] developed mesoporous silica nanoparticles employed as carriers for silver to function as an antibacterial agent against *M. tuberculosis*. Two synthesis methods were employed: a 2D hexagonal mesoporous silica with silver bromide nanoparticles distributed throughout the silica network and a core–shell structure with a metallic AgNP core and a mesoporous silica shell. Both materials exhibited effective in vitro antimycobacterial capabilities, with the minimum inhibitory concentration observed for the silver bromide approach. Also, Ref. [72] designed a dental prosthesis base resin material using 3D printing technology, incorporating acrylate resin modified with a silver-loaded mesoporous silica nanocarrier to improve antimicrobial and mechanical properties. The biocompatibility against oral fibroblasts and antimicrobial effect against *C. albicans* biofilms were studied.

**Figure 11:**
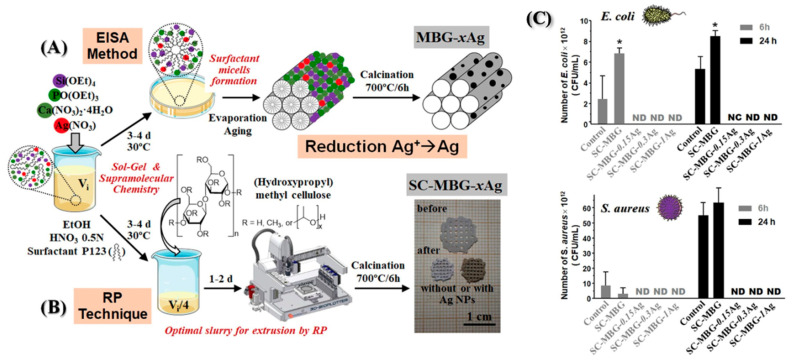
Illustration depicting various synthesis methods for (**A**) MBG-xAg powder materials and (**B**) SC-MBG-xAg scaffolds. (**C**) MTS proliferation assay of pre-osteoblastic cells in the presence of SC-MBG-xAg. Reproduced with modification from reference Sánchez-Salcedo et al. [73], 2023, under the terms and conditions of the Creative Commons Attribution (CC BY) license. https://doi.org/10.1016/j.actbio.2022.10.045.

Moreover, Ref. [73] synthesized 3D-printed scaffolds with a hierarchical pore structure and potent antimicrobial capabilities, suggesting potential utility in bone tissue regeneration (Figure 11). Nanocomposites were created through a one-step sol-gel process, combining mesoporous bioactive glasses (MBG) with metallic AgNPs, utilizing the mesostructure directing agent (P123) and (hydroxypropyl)methylcellulose as a macrostructure template. Evaluations involving pre-osteoblastic cell cultures and bacterial assays (*E. coli* and *S. aureus*) were conducted on the Ag/MBG nanocomposites. As the silver concentration increased, pre-osteoblastic proliferation decreased. Antimicrobial assays revealed a direct correlation between the concentration of Ag-NPs in the MBG matrices and the inhibition of bacterial growth as well as the destruction of biofilms.

Another advance in the biomedical application was the green synthesis approach [74]. The researchers employed Rutin (Ru) extract, a biocompatible flavonoid, as both a reducing agent and a nonsurfactant template for the environmentally friendly synthesis of Ag-decorated mesoporous silica nanoparticles (MSNs). The antimicrobial effectiveness was assessed against *S. aureus*, *E. coli*, and *Candida* strains. The MTT assay performed cytotoxicity testing. Ru-Ag decorated MSNs show antimicrobial efficacy against Gram-positive and Gram-negative bacteria and different fungi, as well as acceptable safety and low cytotoxicity even at low concentrations.

In another attempt to develop a modified approach combining synergistic antibacterial, anti-inflammatory, and pro-vascular strategies for wound healing, Ref. [75] suggested a hydrogel dressing with a double network structure composed of polyethylene glycol diacrylate (PEGDA), catechol-modified hyaluronic acid (C-HA), and a covalent network of Ag-doped mesoporous silica nanoparticles (AMSN). The dual cross-linked configuration of PEGDA/C-HA-AMSN improved the physicochemical characteristics, encompassing tissue adhesion strength, gelation time, and mechanical performance. Outstandingly, PEGDA/C-HA-AMSN functioned as a hydrogel dressing designed to be responsive to the acidic environment of infected wounds by *S. aureus* and *E. coli* bacteria, leading to controllable and optimized Ag delivery, allowing long-lasting antibacterial activity, cytocompatibility, and angiogenesis capacity. Other developments use mixtures of different metals with antimicrobial activity to synergistically increase the positive characteristics desired in biomedical applications. Ref. [76] studied the antibiofilm activity and mechanisms of mesoporous calcium silicate nanoparticles (MCSN) containing varying ratios of silver and zinc, 1:9 and 9:1 were prepared, respectively. The antibiofilm activity was tested on human roots using the *E. faecalis* biofilm model. Ag/Zn-MCSNs exhibited effective antibiofilm activity. Adjusting the Ag and Zn ratio allows for a suitable balance between antibacterial efficacy and cytotoxicity. It is anticipated that Ag/Zn-MCSNs could serve as a novel root canal disinfectant or sealant for root canal treatment.

Moreover, Li and co-workers [77] studied the combination of silver antibacterial capacity and copper angiogenesis-promoting properties. Prepared through a sol-gel method, these MBGNs underwent an in vitro bioactivity assay to assess their capability to form hydroxyapatite in phosphate-buffered saline. Human umbilical vein endothelial cell migration and tube formation assays were conducted to evaluate their angiogenesis-promoting properties. The findings demonstrate that the material exhibits favorable bioactivity, antibacterial capacity, and angiogenesis-promoting properties, positioning it as a multifunctional material with substantial potential in biomedicine.

Other developments focus on using copper ions due to the antibacterial effect [78]. Hosseini et al. [78] synthesized MBGNs doped with copper ions (Cu-PMMBGNs in this contribution). The authors introduced amine functional groups into mesoporous silica nanoparticles, enabling the incorporation of calcium and copper ions without compromising mesoporosity, homogeneity, or nanoparticle shape. The resulting Cu-PMMBGNs were degradable and demonstrated a rapid induction of apatite crystal deposition on their surface within 3 days of immersion in simulated body fluids. Particles with 5 mol % copper ions, at concentrations of 500 and 1000 μg mL^−1^, demonstrated remarkable antibacterial efficacy against *S. aureus*, resulting in a substantial 99.9% reduction in bacterial viability.

**Figure 12:**
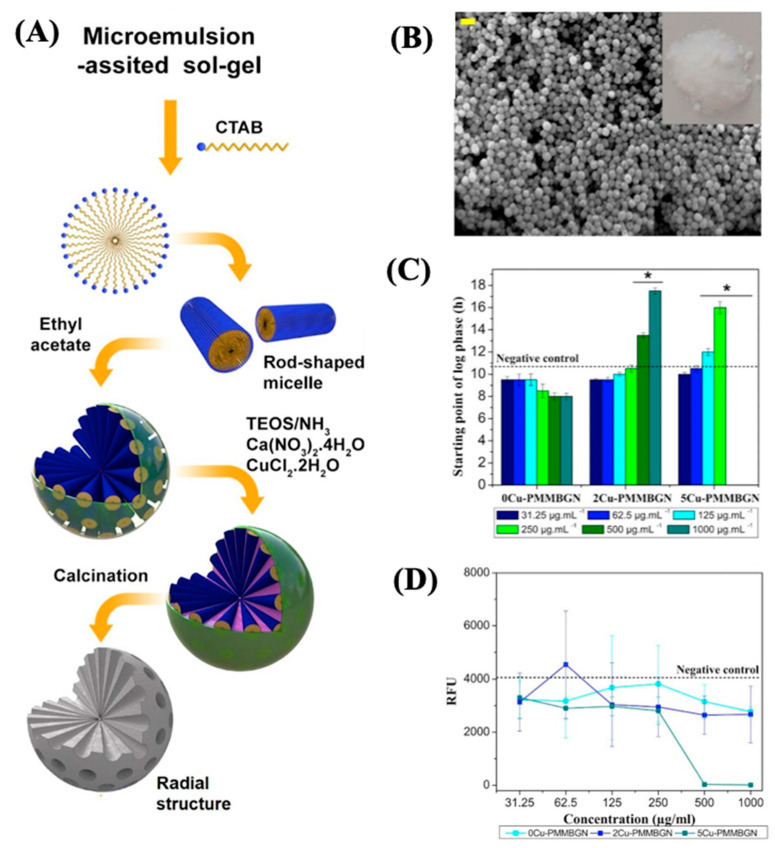
(**A**) Synthesis scheme, (**B**) micrograph, (**C**) effect on the starting point of bacterial growth, and (**D**) MRSA viability of XCu-PMMBGNs. Reproduced with modification from reference Hosseini et al. [78], 2023, under the terms and conditions of the Creative Commons Attribution (CC BY) license. https://doi.org/10.1016/j.bioadv.2022.213198.

Additionally, at a concentration of 500 μg mL^−1^, no notable cytotoxicity was observed towards preosteoblast cells, with cell viability ranging from approximately 85% to 89% compared to the control group. Moreover, their nanoscale dimensions (approximately 100 nm) facilitated their uptake into preosteoblast cells, underscoring their potential as intracellular carriers for combating intracellular bacteria. Refer to Figure 12 for a visual representation of these findings.

Foroutan and co-workers [79] formulated mesoporous glasses based on phosphates (MPG) as promising bioabsorbable materials designed for the controlled release of antibacterial copper ions. MPG was synthesized within the P_2_O_5_-CaO-Na_2_O system, both without doping and doped with 1, 3, and 5 mol% Cu^2+^ ions using sol–gel method and a combination of supramolecular templates and using P123 as a template agent. Over 7 days, controlled release was achieved for phosphates, Ca^2+^, Na^+^, and Cu. The released copper demonstrated a notable reduction in the bacterial viability of *S. aureus* and *E. coli* over 3 days within the MPG.

Other authors studied Zn(II) species as antimicrobial agents, as in [80], who modified the surface of a 3D printed titanium scaffold with Zn-MBG to prevent implant-related infections. The study revealed that as the Zn content increased from 1 mol% to 5 mol%, the specific surface area of MBG decreased from 377.6 m^2^ g^−1^ to 174.5 m^2^ g^−1^, reducing the apatite inducer capacity. Nevertheless, investigations into antibacterial properties indicated an enhancement by adding zinc. Biocompatibility was assessed using MC3T3-E1 cells, revealing that Zn/MBG fosters cell proliferation and exhibits favorable cytocompatibility. Another author used Zn-Loaded SBA-1 and SBA-15 for bone regeneration [81]. A comparison was made regarding the influence of geometry, pore size, and ordered structure of SBA-1 and SBA-15 on zinc loading and release performance. The zinc loading varied between 2.5 and 10 wt% for both mesoporous silicas. Up to 10 wt%, zinc loading had a negligible impact on the texture and morphological properties of the silica. The investigation indicates that SBA-15 demonstrates significantly higher zinc release than SBA-1, enhancing antibacterial activity against Gram-positive and Gram-negative bacteria. A 5 wt% zinc loading is sufficient to exert bactericidal and inhibitory effects on bacterial cells. Samples loaded with 5 wt% zinc induce osteogenic differentiation in avianized bone marrow-derived stromal cells, although SBA-15 samples exhibit superior biocompatibility.

**Figure 13:**
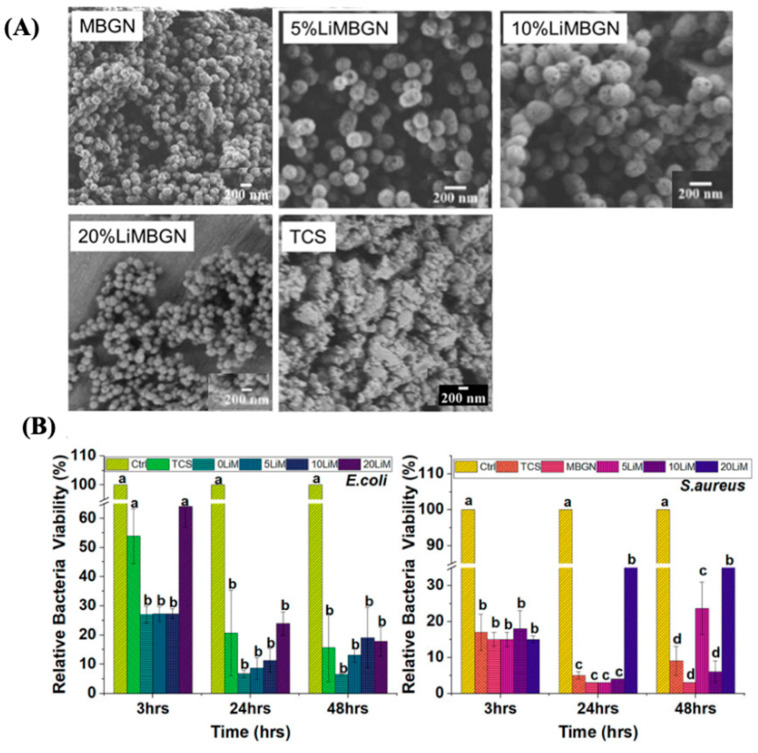
(**A**) Micrographs of MBGNs and TCS. (**B**) The effect of these particles on *E. coli* and *S. aureus*. Reproduced with modification from reference Simila and Boccaccini [82], 2023, under the terms and conditions of the Creative Commons Attribution (CC BY) license. https://doi.org/10.3389/fbioe.2023.1065597.

Other ions were evaluated and used in biomedical applications, such as lithium [82], synthesized and characterized the morphology and chemical structure of tricalcium silicate (TCS) and MBGNs doped with 0%, 5%, 10%, and 20% Li using the sol-gel method. Subsequently, concentrations of 15 mg per 10 mL were incubated in artificial saliva, Hank’s balanced salt solution, and simulated body fluid at 37 °C for 28 days. The study assessed bactericidal effects against *S. aureus* and *E. coli* and potential cytotoxicity against MG63 cells through turbidity measurements. Limited lithium ion incorporation was observed in MBGN. All particles exhibited an alkalizing effect. TCS emerged as AS’s sole particle-forming apatite within 3 days, correlating with higher bioactivity, while MBGN demonstrated superior antimicrobial properties (Figure 13).

Pinto and Souza [83] used cobalt and cerium to find a biomaterial for skin wound healing. Spherical MBGN was synthesized through the sol–gel process, and Ce and Co were introduced into the glass matrix through a post-modification procedure. The release profile of Si, Ce, and Co was tracked over 14 days using simulated exudate fluid. Evaluation of structural properties and biological behavior through various techniques indicated a synergistic effect of Ce and Co on the bioactive glass, leading to samples with elevated cell viability and antibacterial activity.

Table 2 summarizes the various synthesized nanomaterials and their applications in human health.

Na and colleagues presented an approach against antimicrobial infections by combining antibiotics with photothermal therapy using multifunctional nanomaterials [84]. In this study, the inherent photothermal efficiency of two-dimensional rhenium disulfide (ReS_2_) nanosheets was heightened by coating them on mesoporous silica nanoparticles (MSNs) to create a highly efficient light-responsive nanomaterial with controlled-release drug delivery capabilities, known as MSN-ReS_2_. This nanomaterial demonstrated over 99% bacterial killing efficiency against Gram-negative bacteria (*E. coli*) and Gram-positive bacteria (*S. aureus*) upon laser irradiation. These findings highlight the potential of MSN-ReS_2_ as a therapeutic agent with a synergistic bactericidal function. Recently, Song et al. decorated AgNPs in mesoporous silica of SBA-15 coated with melanin-like polydopamine (PDA) as nanocarriers [85]. This composite was loaded with the phytochemical curcumin (CCM) through noncovalent interactions with PDA coatings. The resulting CCM@SBA-15/PDA/Ag composites exhibited favorable biocompatibility and significant inhibitory effect on bacterial growth of Gram-negative *E. coli* and Gram-positive *S. aureus* for 72 and 24 h, respectively, attributed to the bactericidal effects of AgNPs and CCM. This study showcased integrated dual-responsive nanoplatforms for addressing cancer’s infectious bacteria and drug resistance.

Moreover, various types of nanoparticles are under investigation, including those based on MBG, which exhibit favorable structural and textural properties. Sanchez-Salcedo and co-workers explored the regeneration capacity and antibacterial properties of MGNs in the SiO_2_–CaO–P_2_O_5_ system [86]. These assays were evaluated before and after dopping with 2.5% or 4% ZnO and loaded with CCM. The bactericidal action of MGNs with zinc and CCM against *S. aureus* was demonstrated, as a significant reduction in bacterial growth was observed.

**Figure 14:**
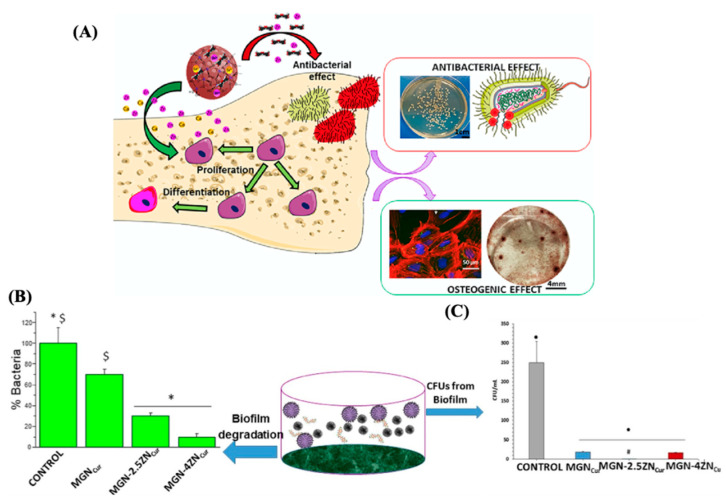
(**A**) Diagram of the antibacterial and osteogenic effects. (**B**) Volumetric views of surface and (**C**) percent of planktonic *S. aureus* CFUs from the biofilm. Reproduced with modification from reference Sánchez-Salcedo et al. [86], 2023, under the terms and conditions of the Creative Commons Attribution (CC BY) license. https://doi.org/10.1016/j.actbio.2023.04.046.

Finally, MC3T3-E1 preosteoblastic cells and *S. aureus* were co-cultured to investigate competitive colonization between bacteria and cells in the presence of MGNs. The results demonstrated the colonization and survival of osteoblasts and the effective inhibition of bacterial adhesion and biofilm formation of *S. aureus* in the co-culture system. The key point of this study was to show the synergistic antibacterial effect of zinc ions combined with CCM, enhancing the bone regeneration characteristics of MGNs containing zinc and curcumin to obtain capable systems for bone regeneration and controlling infection (see Figure 14).

Rama et al. presented an innovative design involving sandwich-like layered mesoporous silica nanofibers doped with Ag and filled with an antibiotic drug, combined with silk fibroin (SF) to enhance synergistic effects [87]. The authors investigated many aspects, including the physicochemical properties of Cephalexin Monohydrate (CEM) within the sandwiched scaffolds through in vitro biomineralization, tensile strength, antibacterial efficacy against *E. coli* and *S. aureus* bacterial strains, in vitro degradation, and osteogenic effects. Incorporating the sandwich technique minimized the faster degradation observed in the monolayered nanofibrous scaffold. Additionally, the combination of CEM/SF embedded in Ag@mesoporous silica sandwich-layered nanofibers exhibited a synergistic antibacterial influence due to the sustained release of drugs. The results suggested that the sandwich technique for nanofibrous scaffolds could be a promising approach for bone regeneration and defect repair. Mu introduced a multifunctional antibacterial coating employing a simultaneous release-inactivation and superhydrophobic anti-adhesion strategy [88]. Mesoporous silica NPs were synthesized and chemically modified in this study. The deposition of nanoparticles created a nanotopography with low surface energy, resulting in a superhydrophobic surface that minimized contact between aqueous bacterial suspensions and surfaces. Simultaneously, the nanopores within the nanoparticles were infused with cinnamon essential oil, gradually diffusing into the surrounding medium and leading to the inactivation of planktonic bacteria. The results demonstrated antibacterial and anti-adhesion properties, reducing the proliferation of *E. coli* and *S. aureus* bacteria by 99.9% and 99.6%, respectively.

García et al. reported MSNs with combined photothermal and antimicrobial capabilities [89]. These nanomaterials acted on bacterial biofilm architecture, inhibiting its growth. This study synthesized core–shell (AuNR@MSN) nanoparticles with PTT properties. Subsequently, nitrosothiol groups with a heat-labile linker were incorporated, enabling enhanced nitric oxide release upon photothermal stimulation with infrared radiation. Moreover, levofloxacin antibiotic was added to make a nanoassembly with therapeutic performance against *S. aureus* biofilms. These results indicated a reduction of approximately 90% in biofilm, demonstrating that localized antimicrobial exposure and PTT improved therapeutic efficacy.

Pamukçu and colleagues proposed an intriguing approach based on core–shell structured nanocomposites for their synergistic antimicrobial effects [90]. They introduced a nanosystem comprising a cerium oxide core and a porous silica shell (CeO_2_@pSiO_2_) that accommodated CCM and lectin. The resulting composite exhibited synergistic antimicrobial effects, particularly against *E. coli*. The study revealed that the mesoporous silica shell surrounding the CeO_2_ core in the nanosystem facilitated the incorporation of CCM and lectin, disrupting bacterial cell motility and increasing the permeability of the inner and outer bacterial cell membranes.

Marinescu and coworkers functionalized SBA-15 with Ru(II) and Ru(III) complexes containing Schiff base ligands derived from salicylaldehyde and amines [91]. These ruthenium complex-loaded SBA-15 silica materials were tested against A549 lung tumor cells and MRC-5 normal lung fibroblasts. The study found that the material containing [Ru(Salen)(PPh_3_)Cl] exhibited the highest antitumoral efficiency, with significant cytotoxicity against cancer cells. Additionally, the antimicrobial study revealed inhibitory effects for all samples, with notable activity against *S. aureus* bacteria. The nanostructured hybrid materials demonstrated potential as active compounds with antiproliferative, antibacterial, and antibiofilm activities.

Ugalde-Arbizu and coworkers synthesized and characterized seven materials based on silica mesoporous nanoparticles functionalized with fluoroquinolones, along with Cu^2+^ or Ag^+^ ions, to assess antibacterial properties against several bacterial strains, including multidrug-resistant strains of *S. aureus* and *P. aeruginosa* [92]. Also, the materials showed antibacterial activity against *S. aureus* and *E. coli*, with Cu^2+^ materials demonstrating increased ROS generation. Ag-based materials exhibited a broader spectrum of activity, inhibiting multidrug-resistant *P. aeruginosa* strains. Moreover, the Ag-based material with a proliferative agent reduced biofilm development and inhibited bacterial growth in a wound-like medium.

Durdu and colleagues developed titanium dioxide-based nanomaterials for dental, orthopedic, and antimicrobial applications [93]. Coatings containing Ag, Cu, and Zn ions on TiO_2_ were produced using micro-arc oxidation and thermal evaporation techniques. The study revealed that Cu-coated surfaces showed better bioactivity properties than those containing Ag. Moreover, Zn-coated surfaces exhibited the best bioactivity. Antibacterial activities against *E. coli* and *S. aureus* were improved by Ag, Cu, and Zn deposition on MAO surfaces, with Ag showing the most effective antibacterial properties [94].

**Figure 15:**
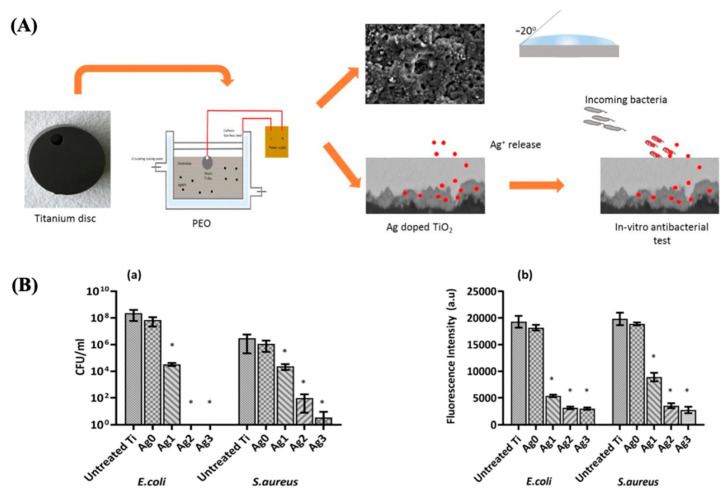
(**A**) Diagram of the procedure to obtain the Ag-doped TiO_2_ coatings. (**B**) Number of CFU (**a**) and metabolic activity (**b**) after 24 h of incubation with different samples. Reproduced with modification from reference Thukkaram et al. [95], 2020, with permission from Elsevier.

Moreover, a recent study demonstrated that Ag-rich antibacterial coatings on TiO_2_ prevent postoperative infections [95], showing excellent antibacterial performance against *E. coli* and *S. aureus* (see Figure 15). Also, Mg-doped TiO_2_ thin films could exhibit antibacterial activity against *S. aureus* [96].

Huang and coworkers used high-power impulse magnetron sputtering to deposit tantalum oxide- and zinc-doped Zn@TaO thin films on Ti with rough and porous surfaces [97]. These coatings exhibited significant antibacterial effects against *S. aureus* and *A. actinomycetemcomitans*. The Zn@TaO-coated films also showed the best antibacterial performance, although they displayed lower cell viability in MG-63 cells, indicating a potential restriction of cell viability.

Hu and colleagues introduced an oxygenated nanocomposite thin film, TaON-Ag. They assessed its antimicrobial properties and effects in an in vitro osteogenic culture model involving rat marrow-derived mesenchymal stem cells (rMSCs) [98]. Additionally, titanium rods coated with TaON-Ag were implanted into a rat femur fracture model, both with and without osteomyelitis, to investigate the nanocomposite’s impact on osteogenesis. The TaON-Ag-coated titanium demonstrated effective antibacterial activity against *S. aureus*, coagulase-negative *Staphylococcus*, *E. coli*, and *P. aeruginosa*. Significantly, the TaON-Ag-coated titanium did not hinder the ossification of rMSCs in vitro or during fracture healing in vivo. The coating also exhibited the ability to inhibit pathogen bacteria adhesion and biofilm formation in both *S. aureus* and *E. coli*. Cabrera and collaborators proposed a strategy to prevent corrosion in metallic implants by applying TiO_2_ films on their surfaces [99]. These films were intended to serve as an extension of the prosthesis to the bone, enhancing biocompatibility. In this work, the authors combined the biocompatible properties of TiO_2_ coatings with the antibacterial activity of metallic silver nanoparticles (AgNPs) by synthesizing thin films on 316 L stainless steel through the sol–gel process. The results indicated a 70% decrease in the corrosion rate for both coated and noncoated samples. Furthermore, the coating was biocompatible with osteoblast cells and exhibited antifungal activity against *A. flavus*.

Widyastuti et al. developed a nanothin film-based antimicrobial coating, TiO_2_/ZnO, using magnetron sputtering and thermal oxidation [100]. The study addressed the antimicrobial activity of TiO_2_/ZnO thin films, showing an increase in the inhibition rate with irradiation time for *E. coli*, *S. aureus*, and *C. albicans*. The effectiveness values were higher than 95%. This research demonstrated that thin films can function as photocatalysts and antimicrobial agents efficiently and cheaply. Moreover, Table 3 summarizes several synthesized nanomaterials and their applications in human health.

## 4. Conclusions and Future Perspectives

The present review highlights the potential of porous materials for bactericidal actions, focusing on both MOFs and mesoporous metallic oxide derivates.

These materials offer versatile opportunities for integration or immobilization across various platforms, enabling them to be processed as readily dispersible solids with highly loading active components. These components can be derived from micro- or mesopores or incorporated into the structure through controlled dissolution.

These materials offer tunability by hosting one or more distinct antimicrobial functions, such as ions, surfactants, and molecular or biomolecular antibiotics. They also present diverse disinfection pathways, including porosity, light-induced ROS generation, or photocatalytic processes. This bi-tunability enhances treatment synergy.

Beyond their intrinsic porosities and expansive surface areas, the controlled manufacture of nanoparticles equips these materials with favorable attributes for applications in biomedicine and health. Notably, their structural stability under ambient and aqueous conditions is crucial for their sustained use in formulations, coatings, and organic polymers. These materials can be integrated with biomolecules to form composites for various applications. Coating their surfaces with organic polymers, for example, improves water dispersion, stability, and biocompatibility.

Also, the construction of mesoscale porous materials represents a rapidly expanding field, encompassing various typologies of materials, including organic, inorganic, crystalline, soft, and amorphous [101,102]. This expansion opens up the potential to develop increasingly specific and advanced antimicrobial platforms [103,104].

However, the broader utilization of these materials faces engineering challenges in materials science, primarily due to the numerous synthetic and functionalization steps that could escalate scaling-up costs. Moreover, current synthetic methods often rely on intricate solvothermal reactions, utilizing toxic and teratogenic organic reagents under high-pressure, high-temperature conditions. This significantly hinders their ability to be produced at an industrial scale and affects their commercial viability. To address this, embracing principles like “green chemical synthesis” is encouraged, involving nontoxic metal sources and biocompatible organic ligands, reducing energy consumption during synthesis, and substituting toxic solvents with water.

Additionally, nanomaterials must be carefully selected based on their properties for specific antimicrobial applications. Factors like biodegradation properties are crucial for medical applications, especially in controlled drug delivery. Surface modifications can enhance their effectiveness and performance in clinical settings, making them indiscernible to the human body. Environmental applications require considerations like separability and reproducibility, and in food applications, designing edible nanostructures can mitigate hazards.

Another critical consideration involves the human cytotoxicity associated with using nanomaterials in indoor air purification or biomedical applications. Evaluating the potential toxicity is crucial, considering safety concerns for nanomaterials. In-depth in vivo toxicity studies and long-term monitoring of tissue accumulation are necessary for future clinical applications, focusing on improving cellular response, biocompatibility, and thermodynamic/kinetic inertness in human cells. Antimicrobial activity studies should extend beyond traditional model strains to include microbial strains relevant to potential applications, such as clinical multidrug-resistant isolates and sterilization of viruses and fungi. Molecular simulation could provide a theoretical foundation for predicting and designing antimicrobial materials, contributing to the comprehension of drug-loading and release kinetics. Combining computational approaches with experimental research would accelerate the development of efficient antimicrobial structures.

Despite reported cases being in a conceptual stage rather than readily deployable materials, exploring these composites for antimicrobial applications is still in its initial phases. Substantial work and research are essential to fulfill the promised potential of these materials, with considerations such as green synthesis, careful material selection, and comprehensive toxicity assessments paving the way for achieving this goal.

## Figures and Tables

**Table 1:** Selection of MOF-based carriers for antimicrobial application.

MOF Carrier	MOF Building Blocks	Antibacterial Component	Mechanism	Effective Against	Ref.
Ceftazidime@ZIF-8	Zn^2+^, 2-H-MeIM	Ceftazidime	Ceftazidime release	*E. coli*	[51]
PCN-224-Ag-HA	Zr^4+^, TCPP	Ag^+^, ROS	Ag^+^ release	*S. aureus*, *MRSA*	[52]
GS5-CL-Ag@CD-MOF	K^+^, γ-CDs	Ag^+^	Ag^+^ release	*E. coli*, *S. aureus*	[53]
ZIF-8-PAA-MB@Ag-NPs@Van-PEG	Zn^2+^, 2-H-MeIM	Ag-NPs, vancomycin, ROS	Ag^+^ and vancomycin release	*E. coli*, *S. aureus*, *MRSA*	[54]
CHX@Cu-BTC	Cu^2+^, H_3_BTC	Cu^2+^, CHX	MOF degradation and CHX release	*E. coli*, *S. aureus*	[55]
Cu-MSA Cu-SA	Cu^2+^, MSA, SA	Cu^2+^	Cu^2+^ release	*E. coli*, *Pseudomona*, *S. aureus*, *B. subtilis*, *C. Albicans*	[56]
Fe/SBA-16/ZIF-8	Zn^2+^, 2-H-MeIM	Zn^2+^	Zn^2+^ release	*P. aeruginosa*, *MRSA biofilms*	[57]
ZIF-8-RF	Zn^2+^, 2-H-MeIM	RF	RF release	*P. aeruginosa*, *MRSA*	[9]
Bio-MOFs	AA, K^+^, Na^+^, Mg^2+^	Azelaic acid	MOF decomposition	*S. aureus*, *S. epidermidis*	[58]
Zn-MOF@curcumin	Zn^2+^, DMA	Zn^2+^, curcumin	Curcumine and Zn^2+^ release	*S. aureus*, *E. coli.*	[59]
MIP-177	Ti^4+^, H_4_mdip	NO	NO release	no reported	[60]

Note: ZIF: zeolitic imidazole framework; 2-H-MeIM: 2-Methylimidazole; γ-CD: gamma-cyclodextrin; TCPP: tetra(4-carboxyphenyl) porphyrin; H_3_BTC: 1,3,5-bencenetricarboxylate; DMA: N,N’-dimethylacetamide; H_4_mdip: 3,3′,5,5′ tetracarboxydiphenylmethane; CHX: chlorhexidine; MSA: mercaptosuccinic acid; SA: succinic acid; RF: rifampicin; AA: azelaic acid.

**Table 2:** Synthesized nanomaterials and their applications in human health.

Nanomaterial	Effective Against	Application	Ref.
Mesoporous silicon nanoparticles	*L. casei*	Wound healing	[67]
Mesoporous silica nanoparticles/AgNPs	*M. tuberculosis*	Antimicrobial	[68]
Core/shell mesoporous silica nanostructures/Nitrogen-sulfur/carbon dots	*S. aureus*, *P. aeruginosa*, *E. coli*	Wound healing	[69]
Ag^+^/mesoporous thin films (SiO_2_ or TiO_2_)	*P. aeruginosa*, *S. aureus*	Long-lasting Antibacterial Thin film coatings	[70]
Ag^+^/SBA-15 or Ag^+^/mesoporous silica	*E. coli*, *P. aeruginosa*, *S. cholerasuis*, *B. cereus*, *S. aureus*	Long-lasting Antibacterial coatings	[71]
Acrylate resin/silver/mesoporous silica nanocarrier	*C. albicans*	Dental prosthesis	[72]
MBG/AgNPs	*E. coli*, *S. aureus*	Bone tissue regeneration	[73]
Ag/mesoporous silica nanoparticles	*S. aureus*, *E. coli*, *Candida*	Biomedical application	[74]
Polyethylene glycol diacrylate/catechol-hyaluronic acid/Ag-mesoporous silica nanoparticles	*S. aureus*, *E. coli*	Wound healing	[75]
Mesoporouscalciumsilicatenanoparticles/silver-zinc	*E. faecalis*	Root canal disinfectant	[76]
80SiO_2_–15CaO–5P_2_O_5_-(5-x)Ag-xCuO (x = 0–5)/MBG	*S. aureus*	Bone formation	[77]
MBGNs/copper ions	*S. aureus*	Antibacterial	[78]
Phosphate-based mesoporous glasses/Cu ions	*S. aureus*, *E. coli*	Bioabsorbable materials	[79]
Zn-MBG	*S. aureus*, *E. coli*	Implants-associated infections	[80]
Zn/SBA-1/SBA-15	*E. coli*, *B. subtilis*	Bone regeneration	[81]
Tricalcium silicate/MBGNs/Li	*S. aureus*, *E. coli*	Biomedical applications	[82]
MBGNs/Ce-Co	*S. aureus*	Wound healing	[83]

**Table 3:** Synthesized nanomaterials and their applications in human health.

Nanomaterial	Effective Against	Application	Ref.
MSN-ReS_2_	*E. coli*, *S. aureus*	Agent therapeutic with a synergistic bactericide function	[84]
CCM@SBA-15/PDA/Ag	*E. coli*, *S. aureus*	Effect of bactericide and drug resistance in cancers	[85]
SiO_2_–CaO–P_2_O_5_	*S. aureus*	Bactericide action and promoting bone regeneration	[86]
Ag-doped mesoporous silica nanofibers	*E. coli*, *S. aureus*	Antibacterial efficacy, bone regeneration, and defect repair	[87]
Modified MSNs	planktonicbacteria, *E. coli*, *S. aureus*	Antibacterial and anti-adhesion properties	[88]
AuNR@MSN	*S. aureus*	Antimicrobial action	[89]
CeO_2_@pSiO_2_	*E. coli*	Bactericide effect	[90]
Ru complex-loaded SBA-15 silica	*S. aureus*, *E. faecalis*	Antitumoral, antiproliferative, antibacterial, and antibiofilm activity	[91]
Modified MSNs	*S. aureus*, *E.* *faecalis*, *E.coli*, *P. aeruginosa*	Antibacterial activity and inhibiting strains of multidrug-resistant *P. aeruginosa*	[92]
Ag-, Cu-, and Zn-based TiO_2_	*E. coli*, *S. aureus*	Antimicrobial activity	[93]
Ti–Cu–N film	*S. aureus*	Antimicrobial activity	[94]
Ag-doped TiO_2_	*E. coli*, *S. aureus*	Antimicrobial activity	[95]
Mg-doped TiO_2_	*S. aureus*, *K. pneumoniae*, *P. aeruginosa*, *E. coli*	Antimicrobial activity	[96]
Tantalum oxide and Ta(Zn)O-coated Ti	*S. aureus*, *A. actinomycetemcomitans*	Antimicrobial activity and surface treatments for titanium-based implants	[97]
TaON-Ag	*S. aureus*, coagulase-negative *Staphylococcus*, *E. coli*, *P. aeruginosa*	Antibacterial activity against common microorganisms in orthopedic infections, demonstrating potential for use in clinical applications	[98]
TiO_2_ films	*A. flavus*	Decrease in the corrosion rate in metallic implants, biocompatible with osteoblast cells, and antifungal activity	[99]
TiO_2_/ZnO	*E. coli*, *S. aureus*, *C. albicans*	Photocatalysts and antimicrobial activity	[100]

## Data Availability

Not applicable.

## References

[1] Dadgostar P. (2019). Antimicrobial Resistance: Implications and Costs. Infect. Drug Resist..

[2] Serwecińska L. (2020). Antimicrobials and Antibiotic-Resistant Bacteria: A Risk to the Environment and to Public Health. Water.

[3] Li Y., Xia X., Hou W., Lv H., Liu J., Li X. (2023). How Effective are Metal Nanotherapeutic Platforms Against Bacterial Infections? A Comprehensive Review of Literature. Int. J. Nanomed..

[4] Li R., Chen T., Pan X. (2021). Metal–Organic-Framework-Based Materials for Antimicrobial Applications. ACS Nano.

[5] Álvarez E., González B., Lozano D., Doadrio A.L., Colilla M., Izquierdo-Barba I. (2021). Nanoantibiotics Based in Mesoporous Silica Nanoparticles: New Formulations for Bacterial Infection Treatment. Pharmaceutics.

[6] Soler-Illia G.J.A.A., Angelomé P.C., Fuertes M.C., Grosso D., Boissiere C. (2012). Critical aspects in the production of periodically ordered mesoporous titania thin films. Nanoscale.

[7] Andersson M., Atefyekta S., Ercan B., Karlsson J., Taylor E., Chung S., Webster T. (2016). Antimicrobial performance of mesoporous titania thin films: Role of pore size, hydrophobicity, and antibiotic release. Int. J. Nanomed..

[8] Pettinari C., Pettinari R., Di Nicola C., Tombesi A., Scuri S., Marchetti F. (2021). Antimicrobial MOFs. Coord. Chem. Rev..

[9] Ahmed S.A., Nur Hasan M., Bagchi D., Altass H.M., Morad M., Althagafi I.I., Hameed A.M., Sayqal A., Khder A.E.R.S., Asghar B.H. (2020). Nano-MOFs as targeted drug delivery agents to combat antibiotic-resistant bacterial infections. R. Soc. Open Sci..

[10] Yan L., Gopal A., Kashif S., Hazelton P., Lan M., Zhang W., Chen X. (2022). Metal organic frameworks for antibacterial applications. Chem. Eng. J..

[11] Fujita D., Suzuki K., Sato S., Yagi-Utsumi M., Yamaguchi Y., Mizuno N., Kumasaka T., Takata M., Noda M., Uchiyama S. (2012). Protein encapsulation within synthetic molecular hosts. Nat. Commun..

[12] Ma M., Chen J., Liu H., Huang Z., Huang F., Li Q., Xu Y. (2022). A review on chiral metal–organic frameworks: Synthesis and asymmetric applications. Nanoscale.

[13] Zha X., Zhao X., Webb E., Khan S.U., Wang Y. (2023). Beyond Pristine Metal–Organic Frameworks: Preparation of Hollow MOFs and Their Composites for Catalysis, Sensing, and Adsorption Removal Applications. Molecules.

[14] Qi X., Shen N., Al Othman A., Mezentsev A., Permyakova A., Yu Z., Lepoitevin M., Serre C., Durymanov M. (2023). Metal-Organic Framework-Based Nanomedicines for the Treatment of Intracellular Bacterial Infections. Pharmaceutics.

[15] Zhang X., Peng F., Wang D. (2022). MOFs and MOF-Derived Materials for Antibacterial Application. J. Funct. Biomater..

[16] Qiao Y., Han Y., Guan R., Liu S., Bi X., Liu S., Cui W., Zhang T., He T. (2023). Inorganic hollow mesoporous spheres-based delivery for antimicrobial agents. Front. Mater. Sci..

[17] Kankala R.K., Han Y.-H., Xia H.-Y., Wang S.-B., Chen A.-Z. (2022). Nanoarchitectured prototypes of mesoporous silica nanoparticles for innovative biomedical applications. J. Nanobiotechnology.

[18] Lei Q., Guo J., Noureddine A., Wang A., Wuttke S., Brinker C.J., Zhu W. (2020). Sol–Gel-Based Advanced Porous Silica Materials for Biomedical Applications. Adv. Funct. Mater..

[19] Vallet-Regí M., Schüth F., Lozano D., Colilla M., Manzano M. (2022). Engineering mesoporous silica nanoparticles for drug delivery: Where are we after two decades?. Chem. Soc. Rev..

[20] Castillo R.R., Vallet-Regí M. (2021). Recent Advances Toward the Use of Mesoporous Silica Nanoparticles for the Treatment of Bacterial Infections. Int. J. Nanomed..

[21] Li B., Liao Y., Su X., Chen S., Wang X., Shen B., Song H., Yue P. (2023). Powering mesoporous silica nanoparticles into bioactive nanoplatforms for antibacterial therapies: Strategies and challenges. J. Nanobiotechnol..

[22] Bellino M.G., Golbert S., De Marzi M.C., Soler-Illia G.J.A.A., Desimone M.F. (2013). Controlled adhesion and proliferation of a human osteoblastic cell line by tuning the nanoporosity of titania and silica coatings. Biomater. Sci..

[23] Pezzoni M., Catalano P.N., Pizarro R.A., Desimone M.F., Soler-Illia G.J.A.A., Bellino M.G., Costa C.S. (2017). Antibiofilm effect of supramolecularly templated mesoporous silica coatings. Mater. Sci. Eng. C.

[24] Moritz M., Geszke-Moritz M. (2015). Mesoporous silica materials with different structures as the carriers for antimicrobial agent. Modeling of chlorhexidine adsorption and release. Appl. Surf. Sci..

[25] Mazzotta E., De Santo M., Lombardo D., Leggio A., Pasqua L. (2022). Mesoporous silicas in materials engineering: Nanodevices for bionanotechnologies. Mater. Today Bio.

[26] Gomez G.E., Soler-Illia G.J.A.A. (2021). Virtual Issue on Multifunctional Nanoporous Materials in Latin America. Chem. Mater..

[27] Batten S.R., Champness N.R., Chen X.-M., Garcia-Martinez J., Kitagawa S., Öhrström L., O’Keeffe M., Paik Suh M., Reedijk J. (2013). Terminology of metal–organic frameworks and coordination polymers (IUPAC Recommendations 2013). Pure Appl. Chem..

[28] Rowsell J.L.C., Yaghi O.M. (2004). Metal-Organic Frameworks: A new class of porous materials. Microporous Mesoporous Mater..

[29] Mac Gillivray L.R., Lukehart C.M., Farrusseng D. (2014). Metal-Organic Framework Materials.

[30] Furukawa H., Cordova K.E., O’Keeffe M., Yaghi O.M. (2013). The Chemistry and Applications of Metal-Organic Frameworks. Science.

[31] Eddaoudi M., Kim J., Rosi N., Vodak D., Wachter J., O’keeffe M., Yaghi O.M. (2002). Systematic Design of Pore Size and Functionality in Isoreticular MOFs and Their Application in Methane Storage. Science.

[32] Zhang W., Ye G., Liao D., Chen X., Lu C., Nezamzadeh-Ejhieh A., Khan M.S., Liu J., Pan Y., Dai Z. (2022). Recent Advances of Silver-Based Coordination Polymers on Antibacterial Applications. Molecules.

[33] Mao F., Su Y., Sun X., Li B., Liu P.F. (2023). Cu(I) Metal–Organic Framework Composites with AgCl/Ag Nanoparticles for Irradiation-Enhanced Antibacterial Activity against *E. coli*. ACS Omega.

[34] Godoy A.A., Bernini M.C., Funes M.D., Sortino M., Collins S.E., Narda G.E. (2021). ROS-generating rare-earth coordination networks for photodynamic inactivation of Candida albicans. Dalt. Trans..

[35] Jing H., Zhao L., Song G., Li J., Wang Z., Han Y., Wang Z. (2023). Application of a Mixed-Ligand Metal–Organic Framework in Photocatalytic CO_2_ Reduction, Antibacterial Activity and Dye Adsorption. Molecules.

[36] Li P., Li J., Feng X., Li J., Hao Y., Zhang J., Wang H., Yin A., Zhou J., Ma X. (2019). Metal-organic frameworks with photocatalytic bactericidal activity for integrated air cleaning. Nat. Commun..

[37] Liu X., Tao Y., Qi K., Chen Z., Qiu Y., Guo X. (2023). Integrated MOF-74 Coatings on Magnesium for Corrosion Control, Cytocompatibility, and Antibacterial Properties. Inorg. Chem..

[38] Motakef-Kazemi N., Ataei F., Dorranian D. (2023). Laser ablation produced graphene/MOF-5 nanocomposite: Antibacterial properties. J. Theor. Appl. Phys..

[39] Duncan M.J., Wheatley P.S., Coghill E.M., Vornholt S.M., Warrender S.J., Megson I.L., Morris R.E. (2020). Antibacterial efficacy from NO-releasing MOF–polymer films. Mater. Adv..

[40] Shetu S.A., Sanchez-Palestino L.M., Rivera G., Bandyopadhyay D. (2022). Medicinal bismuth: Bismuth-organic frameworks as pharmaceutically privileged compounds. Tetrahedron.

[41] Gomez G.E., D’vries R.F., Lionello D.F., Aguirre-Díaz L.M., Spinosa M., Costa C.S., Fuertes M.C., Pizarro R.A., Kaczmarek A.M., Ellena J. (2018). Exploring physical and chemical properties in new multifunctional indium-, bismuth-, and zinc-based 1D and 2D coordination polymers. Dalt. Trans..

[42] Huang R., Zhou Z., Lan X., Tang F.K., Cheng T., Sun H., Cham-Fai Leung K., Li X., Jin L. (2023). Rapid synthesis of bismuth-organic frameworks as selective antimicrobial materials against microbial biofilms. Mater. Today Bio.

[43] Gupta I., Farinas E.T., Mitra S. (2023). Development of carbon nanotube-metal organic framework (MOF) hybrid antiviral microfiltration membrane. Sep. Purif. Technol..

[44] Lee D.N., Gwon K., Kim Y., Cho H., Lee S. (2021). Immobilization of antibacterial copper metal-organic framework containing glutarate and 1,2-bis(4-pyridyl)ethylene ligands on polydimethylsiloxane and its low cytotoxicity. J. Ind. Eng. Chem..

[45] Bhat Z.U.H., Hanif S., Rafi Z., Alam M.J., Ahmad M., Shakir M. (2023). New mixed-ligand Zn(II)-based MOF as a nanocarrier platform for improved antibacterial activity of clinically approved drug levofloxacin. New J. Chem..

[46] Yang J., Yang Y. (2020). Metal–Organic Frameworks for Biomedical Applications. Small.

[47] Wan Y., Xu W., Ren X., Wang Y., Dong B., Wang L. (2020). Microporous Frameworks as Promising Platforms for Antibacterial Strategies Against Oral Diseases. Front. Bioeng. Biotechnol..

[48] Yang M., Zhang J., Wei Y., Zhang J., Tao C. (2022). Recent advances in metal-organic framework-based materials for anti-staphylococcus aureus infection. Nano Res..

[49] Han D., Liu X., Wu S. (2022). Metal organic framework-based antibacterial agents and their underlying mechanisms. Chem. Soc. Rev..

[50] Desai A.V., Vornholt S.M., Major L.L., Ettlinger R., Jansen C., Rainer D.N., de Rome R., So V., Wheatley P.S., Edward A.K. (2023). Surface-Functionalized Metal–Organic Frameworks for Binding Coronavirus Proteins. ACS Appl. Mater. Interfaces.

[51] Sava Gallis D.F., Butler K.S., Agola J.O., Pearce C.J., McBride A.A. (2019). Antibacterial Countermeasures via Metal–Organic Framework-Supported Sustained Therapeutic Release. ACS Appl. Mater. Interfaces.

[52] Zhang Y., Sun P., Zhang L., Wang Z., Wang F., Dong K., Liu Z., Ren J., Qu X. (2019). Silver-Infused Porphyrinic Metal–Organic Framework: Surface-Adaptive, On-Demand Nanoplatform for Synergistic Bacteria Killing and Wound Disinfection. Adv. Funct. Mater..

[53] Shakya S., He Y., Ren X., Guo T., Maharjan A., Luo T., Wang T., Dhakhwa R., Regmi B., Li H. (2019). Ultrafine Silver Nanoparticles Embedded in Cyclodextrin Metal-Organic Frameworks with GRGDS Functionalization to Promote Antibacterial and Wound Healing Application. Small.

[54] Chen H., Yang J., Sun L., Zhang H., Guo Y., Qu J., Jiang W., Chen W., Ji J., Yang Y. (2019). Synergistic Chemotherapy and Photodynamic Therapy of Endophthalmitis Mediated by Zeolitic Imidazolate Framework-Based Drug Delivery Systems. Small.

[55] Soltani S., Akhbari K. (2022). Cu-BTC metal–organic framework as a biocompatible nanoporous carrier for chlorhexidine antibacterial agent. JBIC J. Biol. Inorg. Chem..

[56] Gizer S.G., Sahiner N. (2021). The effect of sulphur on the antibacterial properties of succinic acid-Cu(II) and mercaptosuccinic acid-Cu(II) MOFs. Inorganica Chim. Acta.

[57] Balasamy R.J., Ravinayagam V., Alomari M., Ansari M.A., Almofty S.A., Rehman S., Dafalla H., Rubavathi Marimuthu P., Akhtar S., Al Hamad M. (2019). Cisplatin delivery, anticancer and antibacterial properties of Fe/SBA-16/ZIF-8 nanocomposite. RSC Adv..

[58] Quaresma S., André V., Antunes A.M.M., Vilela S.M.F., Amariei G., Arenas-Vivo A., Rosal R., Horcajada P., Duarte M.T. (2020). Novel AntibacterialAzelaic Acid BioMOFs. Cryst. Growth Des..

[59] Guo C., Cheng F., Liang G., Zhang S., Duan S., Fu Y., Marchetti F., Zhang Z., Du M. (2022). Multimodal Antibacterial Platform Constructed by the Schottky Junction of Curcumin-Based Bio Metal–Organic Frameworks and Ti_3_C_2_T_x_ MXene Nanosheets for Efficient Wound Healing. Adv. Nanobiomed. Res..

[60] Pinto R.V., Wang S., Tavares S.R., Pires J., Antunes F., Vimont A., Clet G., Daturi M., Maurin G., Serre C. (2020). Tuning Cellular Biological Functions Through the Controlled Release of NO from a Porous Ti-MOF. Angew. Chem. Int. Ed..

[61] Yang X.-Y., Chen L.-H., Li Y., Rooke J.C., Sanchez C., Su B.-L. (2017). Hierarchically porous materials: Synthesis strategies and structure design. Chem. Soc. Rev..

[62] Soler-Illia G.J.d.A.A., Sanchez C., Lebeau B., Patarin J., Soler-Illia G.J.A.A., Sanchez C., Lebeau B.P. (2002). Chemical Strategies To Design Textured Materials: From Microporous and Mesoporous Oxides to Nanonetworks and Hierarchical Structures. Chem. Rev..

[63] Ha C.-S., Park S.S. (2019). General Synthesis and Physico-Chemical Properties of Mesoporous Materials.

[64] Park S., Ahn S.H., Lee H.J., Chung U.S., Kim J.H., Koh W.-G. (2013). Mesoporous TiO_2_ as a nanostructured substrate for cell culture and cell patterning. RSC Adv..

[65] Catalano P., Bellino M., Soler Illia J., Costa C., Pizarro R., Pezzoni M. (2017). Use of a Mesoporous Oxide Film in Obtaining a Transparent and Ultra-Thin Ceramic Coating Which Inhibits the Formation of Bacterial Biofilms on the Coated Surface. WO2017191555 (A1). https://nuclea.cnea.gob.ar/items/07822e8a-d5da-4ec2-8bca-bf5664323b9d.

[66] Pezzoni M., Catalano P.N., Delgado D.C., Pizarro R.A., Bellino M.G., Costa C.S. (2020). Antibiofilm effect of mesoporous titania coatings on Pseudomonas aeruginosa biofilms. J. Photochem. Photobiol. B Biol..

[67] Sviridov A., Mazina S., Ostapenko A., Nikolaev A., Timoshenko V. (2023). Antibacterial Effect of Acoustic Cavitation Promoted by Mesoporous Silicon Nanoparticles. Int. J. Mol. Sci..

[68] Montalvo-Quirós S., Gómez-Graña S., Vallet-Regí M., Prados-Rosales R.C., González B., Luque-Garcia J.L. (2021). Mesoporous silica nanoparticles containing silver as novel antimycobacterial agents against Mycobacterium tuberculosis. Colloids Surf. B Biointerfaces.

[69] Pongchaikul P., Hajidariyor T., Khetlai N., Yu Y.-S., Arjfuk P., Khemthong P., Wanmolee W., Posoknistakul P., Laosiripojana N., Wu K.C.-W. (2023). Nanostructured N/S doped carbon dots/mesoporous silica nanoparticles and PVA composite hydrogel fabrication for antimicrobial and anti-biofilm application. Int. J. Pharm. X.

[70] Catalano P.N., Pezzoni M., Costa C., Soler-Illia G.J.d.A.A., Bellino M.G., Desimone M.F. (2016). Optically transparent silver-loaded mesoporous thin film coating with long-lasting antibacterial activity. Microporous Mesoporous Mater..

[71] Irene Litter M., Ahmad A. (2023). Industrial Applications of Nanoparticles.

[72] Aati S., Aneja S., Kassar M., Leung R., Nguyen A., Tran S., Shrestha B., Fawzy A. (2022). Silver-loaded mesoporous silica nanoparticles enhanced the mechanical and antimicrobial properties of 3D printed denture base resin. J. Mech. Behav. Biomed. Mater..

[73] Sánchez-Salcedo S., García A., González-Jiménez A., Vallet-Regí M. (2023). Antibacterial effect of 3D printed mesoporous bioactive glass scaffolds doped with metallic silver nanoparticles. Acta Biomater..

[74] Abbasi M., Gholizadeh R., Kasaee S.R., Vaez A., Chelliapan S., Fadhil Al-Qaim F., Deyab I.F., Shafiee M., Zareshahrabadi Z., Amani A.M. (2023). An intriguing approach toward antibacterial activity of green synthesized Rutin-templated mesoporous silica nanoparticles decorated with nanosilver. Sci. Rep..

[75] Huang L., Li W., Guo M., Huang Z., Chen Y., Dong X., Li Y., Zhu L. (2023). Silver doped-silica nanoparticles reinforced poly (ethylene glycol) diacrylate/hyaluronic acid hydrogel dressings for synergistically accelerating bacterial-infected wound healing. Carbohydr. Polym..

[76] Leng D., Li Y., Zhu J., Liang R., Zhang C., Zhou Y., Li M., Wang Y., Rong D., Wu D. (2020). The Antibiofilm Activity and Mechanism of Nanosilver- and Nanozinc-Incorporated Mesoporous Calcium-Silicate Nanoparticles. Int. J. Nanomed..

[77] Li Y.-J., Wong K.W., Huang Y.-C., Chien C.-S., Shih C.-J. (2023). Evaluation of in vitro bioactivity and angiogenesis-promoting effect for mesoporous bioactive glass codoped with copper and silver. J. Non. Cryst. Solids.

[78] Hosseini M., Hassani Besheli N., Deng D., Lievens C., Zuo Y., Leeuwenburgh S.C.G., Yang F. (2023). Facile post modification synthesis of copper-doped mesoporous bioactive glass with high antibacterial performance to fight bone infection. Biomater. Adv..

[79] Foroutan F., Kyffin B.A., Nikolaou A., Merino-Gutierrez J., Abrahams I., Kanwal N., Knowles J.C., Smith A.J., Smales G.J., Carta D. (2023). Highly porous phosphate-based glasses for controlled delivery of antibacterial Cu ions prepared via sol–gel chemistry. RSC Adv..

[80] Chen Q., Zhao X., Lai W., Li Z., You D., Yu Z., Li W., Wang X. (2022). Surface functionalization of 3D printed Ti scaffold with Zn-containing mesoporous bioactive glass. Surf. Coat. Technol..

[81] Trinh H.T., Tran T.K.A., Arora S., George S.M., Sheri J., Li Z., Yang J., Naruphontjirakul P., Balani K., Karakoti A. (2023). Zn-Loaded SBA-1 and SBA-15 Molecular Sieves for Combined Antimicrobial and Osteogenic Activity. Adv. Mater. Technol..

[82] Simila H.O., Boccaccini A.R. (2023). Sol-gel synthesis of lithium doped mesoporous bioactive glass nanoparticles and tricalcium silicate for restorative dentistry: Comparative investigation of physico-chemical structure, antibacterial susceptibility and biocompatibility. Front. Bioeng. Biotechnol..

[83] Pinto e Souza I.E., Barrioni B.R., Miriceia N.M.L., Sachs D., Ribeiro G.C., Soares D.C.F., Pereira M.M., Nunes E.H.M. (2023). Synergistic effect of cobalt and cerium on the structural properties and biological behavior of sol-gel-derived mesoporous bioactive glass nanoparticles. J. Non. Cryst. Solids.

[84] Na H., Venedicto M., Chang C.-Y., Carrier J., Lai C.-Y. (2023). Infrared-Activated Bactericide: Rhenium Disulfide (ReS2)-Functionalized Mesoporous Silica Nanoparticles. ACS Appl. Bio Mater..

[85] Song Y., Cai L., Tian Z., Wu Y., Chen J. (2020). Phytochemical Curcumin-Coformulated, Silver-Decorated Melanin-like Polydopamine/Mesoporous Silica Composites with Improved Antibacterial and Chemotherapeutic Effects against Drug-Resistant Cancer Cells. ACS Omega.

[86] Sánchez-Salcedo S., Heras C., Lozano D., Vallet-Regí M., Salinas A.J. (2023). Nanodevices based on mesoporous glass nanoparticles enhanced with zinc and curcumin to fight infection and regenerate bone. Acta Biomater..

[87] Rama M., Vijayalakshmi U. (2023). Synergism of silver/CEM drug on novel proteinaceous silk fibroin/mesoporous silica based sandwich-layered nanofibrous scaffolds for osteoregenerative applications. Ceram. Int..

[88] Mu M., Lin Y.-T., DeFlorio W., Arcot Y., Liu S., Zhou W., Wang X., Min Y., Cisneros-Zevallos L., Akbulut M. (2023). Multifunctional antifouling coatings involving mesoporous nanosilica and essential oil with superhydrophobic, antibacterial, and bacterial antiadhesion characteristics. Appl. Surf. Sci..

[89] García A., González B., Harvey C., Izquierdo-Barba I., Vallet-Regí M. (2021). Effective reduction of biofilm through photothermal therapy by gold core@shell based mesoporous silica nanoparticles. Microporous Mesoporous Mater..

[90] Pamukçu A., Karakaplan M.B., Didem Ş.K. (2023). Mesoporous silica shell in a core@shell nanocomposite design enables antibacterial action with multiple modes of action. Nano Futur..

[91] Marinescu G., Culita D.C., Mocanu T., Mitran R.-A., Petrescu S., Stan M.S., Chifiriuc M.C., Popa M. (2023). New Nanostructured Materials Based on Mesoporous Silica Loaded with Ru(II)/Ru(III) Complexes with Anticancer and Antimicrobial Properties. Pharmaceutics.

[92] Ugalde-Arbizu M., Aguilera-Correa J.J., San Sebastian E., Páez P.L., Nogales E., Esteban J., Gómez-Ruiz S. (2023). Antibacterial Properties of Mesoporous Silica Nanoparticles Modified with Fluoroquinolones and Copper or Silver Species. Pharmaceuticals.

[93] Durdu S., Arslanturk A., Aktug S.L., Korkmaz K., Aktas S., Unal F., Yalcin E., Cavusoglu K. (2022). A comparison study on bioactivity and antibacterial properties of Ag-, Cu- and Zn- deposited oxide coatings produced on titanium. J. Mater. Sci..

[94] Aziz I., Mulyani E., Yusuf Y. (2023). Morphological, mechanical and antibacterial properties of Ti–Cu–N thin films deposited by sputtering DC. Heliyon.

[95] Thukkaram M., Cools P., Nikiforov A., Rigole P., Coenye T., Van Der Voort P., Du Laing G., Vercruysse C., Declercq H., Morent R. (2020). Antibacterial activity of a porous silver doped TiO_2_ coating on titanium substrates synthesized by plasma electrolytic oxidation. Appl. Surf. Sci..

[96] Kayani Z.N., Abid H.A., Nazli H., Shahid A., Riaz S., Naseem S. (2023). Mg doped TiO_2_ thin films: Optical, dielectric, photocatalytic, magnetic and antibacterial studies. Mater. Sci. Eng. B.

[97] Huang H.-L., Tsai M.-T., Chang Y.-Y., Lin Y.-J., Hsu J.-T. (2020). Fabrication of a Novel Ta(Zn)O Thin Film on Titanium by Magnetron Sputtering and Plasma Electrolytic Oxidation for Cell Biocompatibilities and Antibacterial Applications. Metals.

[98] Hu C.-C., Chang C.-H., Chang Y., Hsieh J.-H., Ueng S.W.-N. (2020). Beneficial Effect of TaON-Ag Nanocomposite Titanium on Antibacterial Capacity in Orthopedic Application. Int. J. Nanomed..

[99] Cabrera-Rodríguez O., Trejo-Valdez M.D., Torres-SanMiguel C.R., Pérez-Hernández N., Bañuelos-Hernández Á., Manríquez-Ramírez M.E., Hernández-Benítez J.A., Rodríguez-Tovar A.V. (2023). Evaluation of the performance of TiO_2_ thin films doped with silver nanoparticles as a protective coating for metal prostheses. Surf. Coat. Technol..

[100] Widyastuti E., Chiu C.-T., Hsu J.-L., Chieh Lee Y. (2023). Photocatalytic antimicrobial and photostability studies of TiO_2_/ZnO thin films. Arab. J. Chem..

[101] Cautela J., Stenqvist B., Schillen K., Belic D., Månsson L.K., Hagemans F., Seuss M., Fery A., Crassous J.M.J., Galantini L. (2020). Supracolloidal Atomium. ACS Nano.

[102] Piantanida E., Alonci G., Bertucci A., De Cola L. (2019). Design of Nanocomposite Injectable Hydrogels for Minimally Invasive Surgery. Acc. Chem. Res..

[103] Saleemi M.A., Kong Y.L., Yong P.V.C., Wong E.H. (2022). An Overview of Antimicrobial Properties of Carbon Nanotubes-Based Nanocomposites. Adv. Pharm. Bull..

[104] Strokov K., Galstyan A. (2020). Chitosan-Silicon Phthalocyanine Conjugate as Effective Photo-Functional Hydrogel for Tracking and Killing of Bacteria. Eur. J. Org. Chem..

